# In vivo spatiotemporal characterizing diverse body transportation of optical labeled high immunity aluminium adjuvants with photoacoustic tomography

**DOI:** 10.1016/j.pacs.2024.100643

**Published:** 2024-09-07

**Authors:** Fan Meng, Chaohao Liang, Barkat Ali, Changwu Wan, Fengbing He, Jiarui Chen, Yiqing Zhang, Zhijia Luo, Lingling Su, Xiaoya Zhao, Bin Yang, Jian Zhang

**Affiliations:** a The Key Laboratory of Advanced Interdisciplinary Studies, The First Affiliated Hospital of Guangzhou Medical University, Guangzhou Medical University, Guangzhou, Guangdong 510120, PR China; b School of Biomedical Engineering, Guangzhou Medical University, Guangzhou, Guangdong 510182, PR China; c School of Chemical Engineering & Technology, China University of Mining and Technology, Xuzhou, Jiangsu 221000, PR China; d School of Pharmacy, Guangzhou Medical University, Guangzhou, Guangdong 510182, PR China

**Keywords:** Photoacoustic imaging, Aluminium adjuvant, Lymph vessels, Vaccine, Transport phenomena

## Abstract

Vaccine development requires high-resolution, in situ, and visual adjuvant technology. To address this need, this work proposed a novel adjuvant labeling that involved indocyanine green (ICG) and bovine serum albumin (BSA) with self-assembled aluminium adjuvant (Alum), which was called BSA@ICG@Alum. This compound exhibited excellent photoacoustic properties and has been confirmed its safety, biocompatibility, high antigen binding efficiency, and superior induction of immune response. Photoacoustic tomography (PAT) tracked the distribution of Alum in lymph nodes (LNs) and lymphatic vessels in real time after diverse injection modalities. The non-invasive imaging approach revealed that BSA@ICG@Alum was transported to the draining LNs 60 min after intramuscular injection and to distal LNs within 30 min after lymph node injection. In conclusion, PAT enabled real-time three-dimensional and quantitative visualization, thus offering a powerful tool for advancing vaccine design by providing critical insights into adjuvant transport and immune system activation.

## 1 Introduction

Vaccinations are a cornerstone in safeguarding public health by actively bolstering the body's resistance to infectious agents [1], [2], [3]. Adjuvants, particularly aluminium adjuvant (Alum), enhance and modulate immune reactions, and have gained approval from the Food and Drug Administration (FDA) for use in human vaccines. Alum's inclusion in vaccines combating diseases ranging from pertussis to the novel coronavirus emphasizes the importance of researching its broader applications [4], [5], [6], [7], [8]. The extensive utilization of Alum underscores the necessity for further research into its potential applications in a diverse range of vaccines. Following injection, the vaccine initiates a cascade of events within the body's lymphatic system, facilitating the transportation of antigens from the periphery to the draining lymph nodes (LNs) [9], [10]. The lymphatic system plays a core role in capturing antigens and directing them to the immune system's hubs, which bolsters vaccination efficacy [11], [12], [13]. Additionally, visualizing vaccine distribution within the lymphatic system is essential for comprehending how vaccines stimulate immune response [14], [15], [16]. Finally, tracing vaccine passage through the body can inform modifications to vaccine formulation, dosage, or delivery to enhance effectiveness and safety [17], [18].

In recent years, the empirical approach to vaccine evaluation has impeded the refinement of vaccine efficacy. Although imaging technologies [19], [20], [21], [22], [23] such as computed tomography (CT) and magnetic resonance imaging (MRI) provide anatomical details [24], [25], and positron emission tomography (PET) scans offer functional insights [26], these methods have limitations, including invasive procedures and poor dynamic tracking capability. Vaccine visualization necessitates not only high-resolution images to facilitate tracking during tissue transport [27], [28] but also to track the vaccine's adjuvant markers in a manner that avoids the possibility of altering their original biodistribution properties and potential interference and damage to the tissue [29]. Therefore, security and real-time high-resolution monitoring of vaccine dynamics to track the spatiotemporal transport of organisms remains a challenge. Photoacoustic imaging (PAI) is non-invasive and has broad application prospects in biomedicine [30]. It has garnered significant utility in tumor therapy, angiogenesis, drug delivery, lymph vessels (LVs), skin and beyond [31], [32], [33], [34], [35], [36], [37], [38], [39]. They draw upon the optical absorption coefficient of imaging tissue and the administration of contrast agents. The most commonly utilized contrast agent is indocyanine green (ICG), a dye approved by the FDA for *in vivo*. This technology offers the capability of achieving safety, high contrast, high resolution, and depth visualization for the penetration of biological tissues [40], [41], [42], [43]. PAI offers a novel approach to evaluating vaccine transport and developing strategies for vaccine development.

**Fig. 1:**
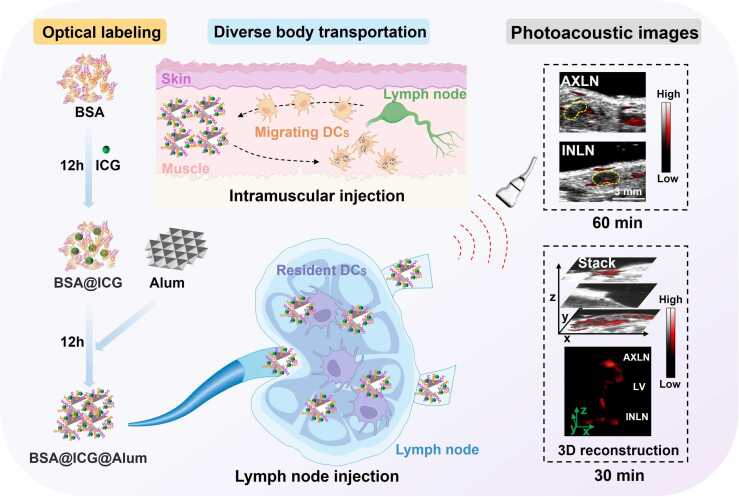
The developed BSA@ICG@Alum suspension features ICG molecules coupled to BSA amalgamated with Alum. PAI was used to visualize INLN to AXLN transport and immune activation following intramuscular injection in mice. In addition, PAI dynamically visualized transport and immune response following lymph node injection, specifically tracking adjuvants imaged in the LV, highlighting the potential of this formulation for targeted vaccine delivery and robust immune response.

In this study, ICG interacted with bovine serum albumin (BSA) by noncovalent bonding to form BSA@ICG complexes, as previously described in reference [44]. Subsequently, driven by electrostatic interaction, Alum adsorbed to BSA, thereby forming the BSA@ICG@Alum structure (Fig. 1). The complex was utilized in the present study and administered via intramuscular and lymph node injection. The distribution and accumulation of Alum in the inguinal lymph node (INLN) and axillary lymph node (AXLN), as well as the transport behavior in the lymphatic vessel (LV) after injection, were monitored using photoacoustic tomography (PAT). Furthermore, this study traced the dynamic transport of the BSA@ICG@Alum complex from the INLN to the AXLN and revealed the overall distribution of Alum in the entire lymphatic system through the use of three-dimensional (3D) reconstruction of photoacoustic (PA) signals. Finally, the dynamics of Alum transport in the lymphatic system at different time points for both intramuscular and lymph node administration modes were examined, with a particular focus on the temporal efficiency of Alum transport to the draining LNs and distal LNs. Specifically, we explored whether Alum, a substantial particulate adjuvant, can be trafficked efficiently through the lymphatic network post-injection, providing valuable insights for further research and the development of immunological interventions.

## 2 Materials and methods

### 2.1 Materials

All chemical reagents were sourced commercially and not subjected to additional purification unless specified. Aluminium hydroxide adjuvant (Imject™ Alum Adjuvant, 77161) was purchased from Invitrogen, USA. ICG (S46424) and Cyanine5 (Cy5) (S25173) were purchased from Yuanye, China. Lumogallion (Ga) was purchased from Shanghai Macklin Biochemical Technology Co., Ltd, China. Macrophages (RAW264.7) were purchased from Wuhan Pricella Biotechnology Co., Ltd, China. BSA (A66421) and phosphate-buffered saline (PBS) (pH 7.2–7.4, 0.01 M) were purchased from ACMEC, China. Fetal Bovine Serum (FBS) and Cell Counting Kit-8 (CCK-8) (FC101–02) were purchased from TransGen Biotech, China. Dulbecco's modified eagle medium (DMEM) (C11995500bt) and Trypsin-EDTA (0.25 %, 25200056) were purchased from Gibco, USA. Penicillin-Streptomycin Solution (100×, BsAb, BL505A) was purchased from Biosharp, China. Ovalin (OVA) was purchased from NJDULY, China. The Immunoglobulin G (lgG) ELISA kit (OVA, RX-D203302M) was purchased from Ruixin, China. Isoflurane (R510) was purchased from Sigma-Aldrich, USA. PE/Cyanine 7 anti-mouse CD11c Antibody was purchased from BioLegend, USA. Deionized water was distilled in the experiments.

### 2.2 Synthesis of BSA@ICG@Alum

The solution was prepared by dissolving 47 mg of BSA in double distilled water (15 mL, ddH_2_O) and 8 mg of ICG in 5 mL of Dimethyl sulfoxide (DMSO). The ICG solution was gradually added to the BSA solution and stirred in the dark for 12 h. After the reaction, dialysis was performed for 24 h, followed by 5 mL of the BSA@ICG solution taken for further use. The remaining solution was added to 500 μL (20 mg, aluminium hydroxide) and diluted Alum into a 4 mL solution. BSA@ICG@Alum suspension was obtained by stirring in the dark for 12 h and undergoing dialysis for 24 h.

### 2.3 Characterization of BSA@ICG@Alum

Scanning electron microscope (SEM) images were recorded using a Phenom (Thermo Scientific Apreo 2 SEM, Thermo Fisher Scientific, USA) at 10 kV, and element distribution was determined using an Energy Dispersive Spectrometer (OXFORD ULTIM Max65, Thermo Fisher Scientific, USA) (EDS). Size and zeta potential were performed using Dynamic Light Scattering (ZETASIZER NANO ZS, Malvern Panalytic, England) (DLS). The Ultraviolet-visible spectroscopy (UV-Vis) absorption and fluorescence spectra (200 μg/mL) of ICG alone, Alum alone, and BSA@ICG@Alum were recorded using an ultraviolet (UV) spectrophotometer (Evolution 300, Thermo Scientific, USA) and a fluorescence spectrometer (FLS1000, Edinburgh Instruments Ltd, England), respectively. The maximum absorption peak was analyzed and compared to explore the interaction between BSA@ICG@Alum and ICG. Centrifugation of Alum and BSA@ICG@Alum liquids (4 mg/mL), taking the supernatant, measuring the content of aluminium by Inductively Coupled Plasma (ICP) (ICPMS-2030LF, Shimadzu, Japan), and comparing the rate of exudation.

### 2.4 Cytotoxicity of BSA@ICG@Alum

CCK-8 assessed the cellular toxicity of BSA@ICG@Alum exposure. Macrophage suspension was inoculated into 96-well plates and incubated at 37 ℃ for 24 h under experimental conditions. Wells were supplemented with varying concentrations (0, 33.6, 67.2, 134, 538, and 1076 μg/mL) of BSA@ICG@Alum solution. The cells were subsequently cultured in an incubator for 24 h and 48 h. Wells were treated with CCK-8 solution (10 μL) and incubated for 2 h, and the absorbance at 450 nm was determined using a microplate reader (Spark 10 M, Tecan, Switzerland).

### 2.5 Antigen adsorption rates of OVA for BSA@ICG@Alum

OVA protein (20 μg) as antigen was reacting with Alum (40 μg) and BSA@ICG@Alum (40 μg) for 30 min. The mixture was centrifuged at 1000 × g for 10 min, and the supernatant was collected. The microprotein Bicinchoninic acid (BCA) (Beyotime, China) method was used to determine OVA's antigen adsorption rates for Alum and BSA@ICG@Alum. Added 10 μL solution to a 96-well enzyme label plate, added 250 μL working liquid, mixed, and incubated at 37 ℃ for 30 min. The microplate reader label measured absorbance at 562 nm. The protein antigen adsorption rate was calculated using the formula:

Antigen adsorption rate = (OVA - OVA in the supernatant)/OVA × 100 %.

### 2.6 Laser confocal characterization of antigen adsorption

Cy5 was conjugated with the OVA model antigen, and Cy5 was slowly added to the OVA solution, shaken, and incubated at room temperature for 60 min. Gently strew the EP tube for 10–15 min to the mix reagents and improve labeling effectiveness. Ga was used to label aluminium. Dissolve Ga in acetic acid buffer and incubate with BSA@ICG@Alum in darkness for 60 min. Samples were washed twice with an acetic acid buffer solution for 15 min. Ga-labeled aluminium solution was diluted ten times and mixed with Cy5-OVA for over 30 min. The adjuvant-antigen combination was diluted tenfold with water. Dilute 10 μL liquid on the slide, use 20× lens for view, followed by 100× oil lens. Choose an appropriate fluorescent channel, designate green for Alum and red for OVA protein antigen. Ga has 485 nm excitation and 598 nm emission wavelength. The excitation and emission wavelengths of Cy5 were 649 nm and 670 nm. Cy5 adsorption was observed using a fluorescence microscope (BZ-X800E, KEYENCE, Japan).

### 2.7 Antigen retention effect of BSA@ICG@Alum

To estimate the antigen retention effect at the injection site, OVA antigens used Cy5 labeling and were loaded with Alum and BSA@ICG@Alum, respectively. Kunming mice (male and female, age 8–10 weeks, weight 35–45 g) were injected with OVA (50 μL, 20 μg), OVA/Alum (50 μL, 20 μg OVA and 40 μg Alum), and OVA/BSA@ICG@Alum (50 μL, 20 μg OVA and 40 μg BSA@ICG@Alum) intramuscularly. The antigen library effect was assessed using fluorescence attenuation at the injection site and tracked using an *in vivo* imaging system (PerkinElmer IVIS Lumina LT, Perkin Elmer, USA). Fluorescence imaging was set as wavelengths: 649 nm for excitation and 666 nm for emission. The ethical clearance for this study was provided by the Institutional Animal Care and Use Committee of Guangzhou Medical University (GY2023–218).

### 2.8 Determination of the concentration of IgG in serum

Alums' humoral immunity was discussed before and after ICG labeling, as they were only attached to cell membrane surfaces. Humoral immunity was tested using OVA as a model antigen in mice injected intramuscularly on days 0, 14, and 28 for humoral responses. Three injections of [OVA only (20 μg), OVA/Alum (20 μg OVA, 40 μg Alum), OVA/BSA@ICG@Alum (20 μg OVA, 40 μg BSA@ICG@Alum), Alum only (40 μg), BSA@ICG@Alum only (40 μg)] were performed, with orbital blood extracted to collect serum samples on day 29. For the ELISA analysis of IgG, 50 μL of the sample was added to a 96-well plate, incubated for 30 min, washed with washing liquid, and added peroxidase-labeled detection antibody. The substrate mixture was mixed, and the absorbance of each well was read on a microplate reader.

### 2.9 Histological analysis

Serum was collected on day 29, mice were euthanized, and organs (heart, liver, spleen, lung, and kidney) were extracted and fixed in 4 % paraformaldehyde. Following paraffin embedding, tissue segments were stained, and images were captured by a digital pathology slide scanner (Aperio CS2, Leica, Germany) for analysis.

### 2.10 Parameters of PAT

Small animal PAI system (Vevo®·LAZR-X, FUJIFILM VisualSonics, Canada) (Sampling Rate: 128 MHz; Photoacoustics Frame Rate (max): 20 fps), equipping YAG laser with an optical parametric oscillator (OPO) for wavelength tuning with a second harmonic generator (Laser Pulse Rate: 20 Hz; Wavelength Step Size: 1 nm; Wavelength Tuning Speed: < 0.4 sec; Peak Energy: 26 mJ at 680 nm; 30 MJ at 970 nm; Axial Resolution: 50 µm; Lateral Resolution: 110 µm; Bandwidth: 20–46 MHz; Transducer frequency: 33 MHz; Image Width (max): 15.4 mm; Penetration Depth: 1–2 cm; System Dynamic Range: 70 dB).

### 2.11 PA properties of BSA@ICG@Alum

Using the Vevo® Spectro program, the imaging wavelength was selected based on the spectrum of the intended material for PA spectroscopy. Identify signals on the ultrasound image and map the region of interest. *In vitro* PA properties of BSA@ICG@Alum were detected using laser-tuned agar samples at 680–960 nm, determining the optimal wavelength for the PA signal. A simulation of optical absorption, scattering, and other parameters of human tissues was conducted using agar, agar sample preparation (a mold was printed with a 3D printer with an aperture in the middle set to hold the sample, with a diameter of 5 mm), the sample was diluted in advance with agar solution to a concentration of 150 μg/mL, 100 μg/mL, 50 μg/mL, 20 μg/mL, and water, and the samples were filled to the full aperture in the solution state, and cooled at room temperature away from light, and then scanned with a PA imager longitudinal section, selecting 130 × 130 pixels and cropping to obtain a PA image. The change in PA signal intensity of BSA@ICG@Alum (150 μg/mL) within 20 min was measured at a wavelength of 800 nm using the same method. Place ICG (150 μg/mL) and BSA@ICG@Alum (150 μg/mL) with a 0.5 mm capillary tube on chicken breasts of varying thickness. A small animal PA system recorded changes in the area and signal intensity with depth at 800 nm. The area was quantified using ImageJ (1.54 f-win64, National Institutes of Health, USA), and the PA signals were extracted by summing the red pixel values of the circled area using MATLAB (R2023a, MathWorks, USA) code and dividing by the circled area to obtain the average PA signal intensity.

### 2.12 PAT monitored the transport of mice

BSA@ICG@Alum (50 μL, 800 μg/mL) was injected into the gluteal muscle and INLN, respectively. Pre-injection, 30 min, 60 min, and 120 min later, mice were anesthetized with isoflurane, and a small animal PAI system was used to image them at 800 nm. Imaging sites included the gluteal muscle, the AXLN, the LV, and the INLN. MATLAB was used to analyze PA signal values quantitatively. To gather dynamic PA images, a stepper motor-driven platform was created to capture fixed animal body images. This platform enabled mice to perform XY motion, the step size (0.3 mm) used and the total scanning distance of 50 mm, capturing dynamic PA images. The time resolution was 0.21 s, and the imaging speed was 0.05 m/s. The total difference before and after tracking the dynamic transport of the adjuvant was 10 s, implying that there was a 10-second delay between the acquisition of the signal from the distal lymph node and the time of injection. The MATLAB software segments video and the ImageJ software images and quantitates for 3D imaging. PA signal quantification was consistent with *in vitro* and quantified by MATLAB extraction.

### 2.13 Aluminium in LNs was detected by ICP

We injected BSA@ICG@Alum into the INLN of mice, and after 30 min, we took the AXLN and INLN of the mice, nitrolysis with aqua regia for 48 h and used ICP to measure the presence of aluminium.

### 2.14 Dendritic cells enrichment in draining LNs

The study involved mice administered intramuscular and lymph node injection at three distinct time points (days 0, 1, and 3), the draining LNs of the mice were removed, and the tissues were ground to homogenate. The cell suspension was filtered through a 100-mesh nylon mesh, and the precipitate was centrifuged at 300 × g for 5 min, and the supernatant was discarded. The cell suspension was washed twice by centrifugation with PBS containing 1 % BSA, and the cell concentration (1 × 10^7^ cells) was adjusted with PBS containing 1 % BSA and set aside for use. A fluorescent labeling antibody (PE Cyanine7-CD11c) was added, mixed well, and incubated at 4 ℃ without light for 30 min. Add PBS containing 1 % BSA to resuspend the cells, centrifuge the cell suspension at 300 × g for 5 min, discard the supernatant, add 200 μL of PBS containing 1 % BSA to resuspend the cells, and then detect and analyze the cells by flow cytometry (CytoFLEX S.4, Beckman Coulter Inc., USA).

### 2.15 Statistical analysis

The data were expressed as means ± standard deviation (SD), and the sample numbers were indicated in the corresponding figure captions. Statistical differences were determined by the student's t-test using GraphPad Prism software. Statistical significance was set at p < 0.05.

## 3 Results and discussion

### 3.1 Characterization of BSA@ICG@Alum

**Fig. 2:**
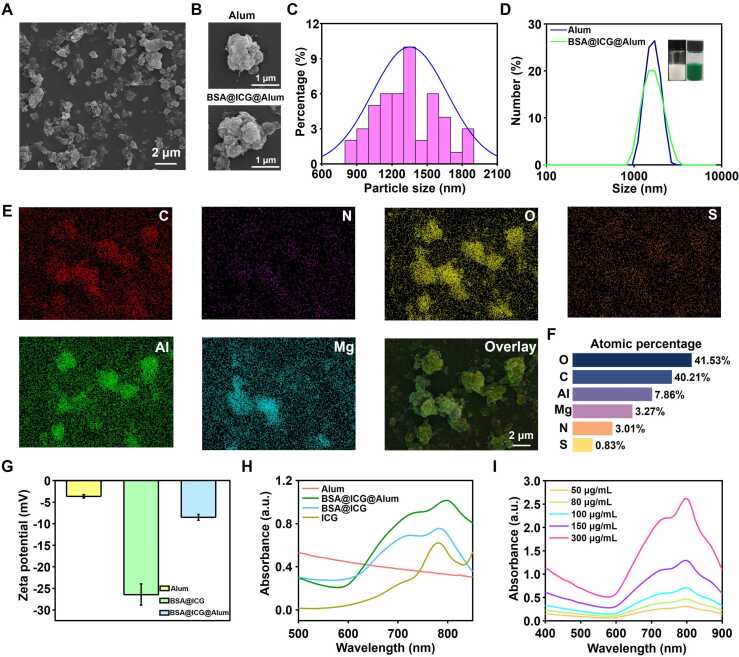
Characterization of BSA@ICG@Alum. (A) SEM image of BSA@ICG@Alum. (B) SEM images of a single Alum particle and BSA@ICG@Alum particle. (C) The particle size distribution of BSA@ICG@Alum by SEM. (D) Size distribution of BSA@ICG@Alum measured by DLS. (E) EDS distribution of BSA@ICG@Alum different elements. (F) The proportions of the various elements of BSA@ICG@Alum. (G) Zeta potential of BSA@ICG@Alum. (H) UV absorption spectra of ICG and BSA@ICG@Alum. (I) UV absorption spectra of BSA@ICG@Alum at different concentrations.

ICG, BSA, and Alum were combined in self-assembly to form BSA@ICG@Alum suspension. This suspension maintained uniform consistency even after storage in various liquid systems (Fig. S1). SEM characterization compared the morphology changes of BSA@ICG@Alum and non-modified Alum. Commercial Alum was non-uniform in shape and had a 710 nm to 1410 nm particle diameter (Fig. S2A, B), whereas BSA@ICG@Alum indicated the shape is irregular (Fig. 2A, B) and a statistical distribution concentrated on 900 nm to 1800 nm (Fig. 2C). Furthermore, DLS analysis indicated a 1638 nm particle size in BSA@ICG@Alum, thus corroborating the SEM observations (Fig. 2D). To investigate the distribution of ICG and BSA within the BSA@ICG@Alum complex, it was observed that aluminium and magnesium were the main components of Alum, while BSA and ICG contain oxygen, nitrogen, and sulfur elements (Fig. 2E). Elemental analysis indicated a homogeneous distribution of ICG and BSA within the Alum, indicating uniform distribution. Aluminium accounts for 7.86 % of the sample, lower than the unlabeled Alum (25.14 %), indicating that other elements are added to the complex. After the Alum was labeled, the proportion of oxygen decreased from 61.47 % to 41.53 % (Fig. 2F, S2C, and S2D). This demonstrated that a portion of the oxygen in the Alum was no longer present on the surface, and the Alum was enveloped by BSA@ICG, indicating successful labeling with ICG. After labeling the Alum, their electrostatic adsorption effect was examined. Commercial Alum has −3.24 mV, while BSA@ICG has −23.8 mV. Since the natural surface of the Alum adjuvant particles contains a neutral bias negative charge, the adsorption of BSA@ICG can mask the original potential, causing BSA@ICG@Alum to have a more negative zeta potential. Therefore, the zeta potential results indicate that BSA@ICG@Alum has a more negative potential. The zeta potential of BSA@ICG@Alum was −7.8 mV (Fig. 2G). The absolute value of the zeta potential of the labeled Alum indicated a definite elevation, and the particle exhibited more excellent stability.

Moreover, the zeta potential of BSA@ICG@Alum was lower than that of the Alum due to the BSA@ICG, demonstrating the effectiveness of the Alum and BSA@ICG combination. Refine UV profiles for samples containing varying ICG levels (Fig. 2H), and it is evident that samples containing ICG indicated distinct peaks in the range of 780–800 nm, indicating a successful introduction of ICG. UV spectral results indicated a peak increasing with increasing concentration of BSA@ICG@Alum in solutions, confirming the successful amalgamation of ICG and Alum (Fig. 2I). To verify whether BSA@ICG@Alum leaches Al^3+^ affects safety in body, We use ICP to detect aluminium leakage rate. The results (Fig. S3A, B) showed that the leakage rate of Alum was 0.043 %, and that of BSA@ICG@Alum was 0.016 %, which was not significantly different after comparison. Moreover, the combination of BSA@ICG@Alum with BSA@ICG was closer, resulting in a lower leakage rate. Therefore, although BSA@ICG@Alum contains aluminium, it does not affect the safety in body.

### 3.2 Biocompatibility and immunological characterization

**Fig. 3:**
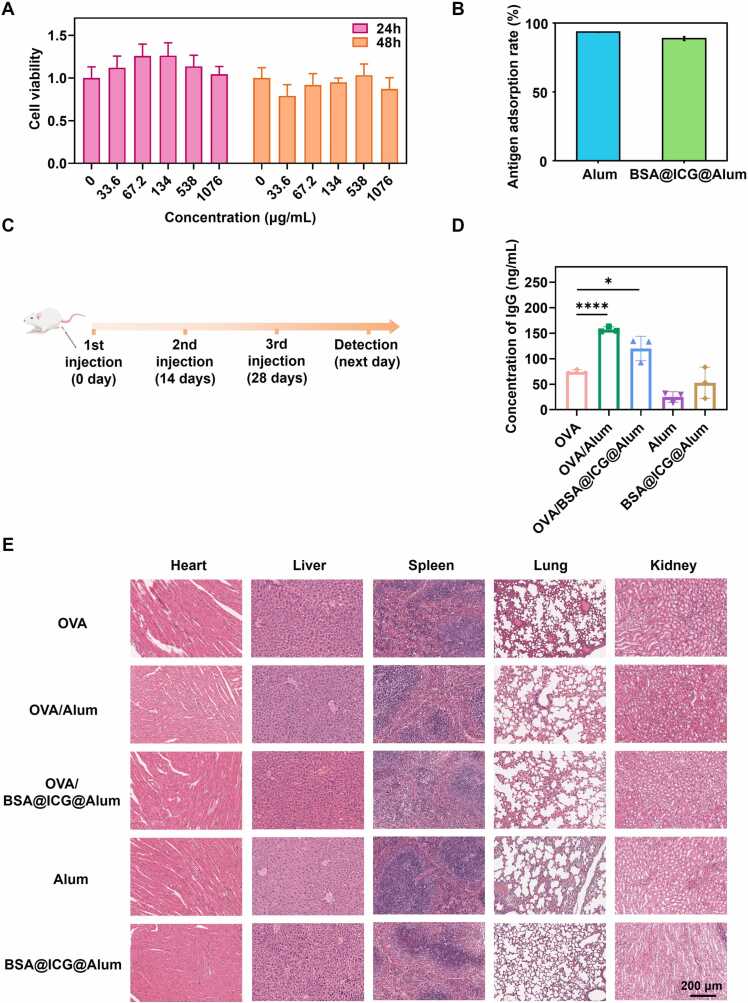
Biocompatibility and immunological characterization. (A) Cytotoxicity of BSA@ICG@Alum. (B) Antigen adsorption rates of OVA for Alum and BSA@ICG@Alum. (C) Temporal dimension of intramuscular injection in mice. (D) The concentration of OVA-specific IgG in serum (Collected on day 29 after three injections). Data were shown as mean s.e.m (n = 3), *P < 0.05, ****P < 0.0001. (E) H&E staining of vital organ sections in mice.

The above results indicated that BSA@ICG@Alum displays a compelling combination. However, further investigation was needed to evaluate safety *in vivo*. Subsequently, incubation of cells with BSA@ICG@Alum for 24 h and 48 h indicated a higher cell survival rate, with cell viability exceeding 80 % even at high concentrations. These results suggested that BSA@ICG@Alum exhibits exceptional biocompatibility (Fig. 3A). Since the adjuvant itself was not antigenic and must be injected into the body beforehand or mixed with antigens to enhance the immune response to antigens, it was necessary to demonstrate the effectiveness of BSA@ICG@Alum as an adjuvant in adsorbing antigens using OVA as the model antigen. Both Alum and BSA@ICG@Alum exhibit near 100 % adsorption rates, enhancing immune response to specific antigens (Fig. 3B). To assess the surface antigen adsorption efficiency, Cy5-labeled antigens and Ga-labeled BSA@ICG@Alum were co-located to assess surface antigen adsorption efficiency, demonstrating the high antigen absorbability of the prepared BSA@ICG@Alum (Fig. S4). *In vitro*, studies indicated that BSA@ICG@Alum exhibited favorable biocompatibility and antigen adsorption rates. To verify the *in vivo* antigen retention effect as an adjuvant, OVA antigen was used as a control. The OVA/Alum group demonstrated a more durable and potent antigenic effect within 12 h. The OVA/BSA@ICG@Alum group demonstrated a more durable and potent antigenic effect under Alum compared to the OVA group after 24 h (Fig. S5A, B), and the fluorescence intensity and antigen retention of BSA@ICG@Alum were found to be comparable to those of Alum after 24 h. It was evidenced that BSA@ICG@Alum remained at the injection site for an extended period, enhancing the efficiency of interaction between antigen-bearing and antigen-presenting cells and creating a prolonged immune response under favorable conditions.

Alum stimulates humoral immune responses, and serum IgG was a commonly employed method for evaluating humoral immune function [45], [46]. Consequently, the concentration of specific IgG in serum was quantified to assess the impact of BSA@ICG@Alum on humoral immunity. Serum was collected from mice following three intramuscular injections, administered two weeks apart (Fig. 3C). Subsequently, serum samples were collected on the 29th day, and the concentration of IgG antibodies was quantified (Fig. 3D). When combined with the OVA antigen, Alum significantly increased the concentration of lgG antibodies by 2.12 times compared to the concentration observed in the OVA antigen alone. The OVA/BSA@ICG@Alum group exhibited a 1.41-fold increase in lgG concentration compared to the OVA antigen alone, indicating a significant difference. The results demonstrated that the OVA antigen, when combined with Alum or BSA@ICG@Alum, elicited a more robust immune response than the OVA antigen alone, demonstrating the adjuvant properties of the optically labeled adjuvant.

To ascertain the safety profile of BSA@ICG@Alum, following the administration of a humoral immunization to mice, their significant organs were harvested and subjected to histological examination using hematoxylin and eosin (H&E) staining (Fig. 3E) revealed that no discernible morphological damage or inflammation was observed in the “OVA only”, “OVA/Alum”, “OVA/BSA@ICG@Alum”, “Alum only”, and “BSA@ICG@Alum” groups. These results indicated that the material was of low toxicity and high biocompatibility.

### 3.3 *In vitro* PAI of BSA@ICG@Alum

**Fig. 4:**
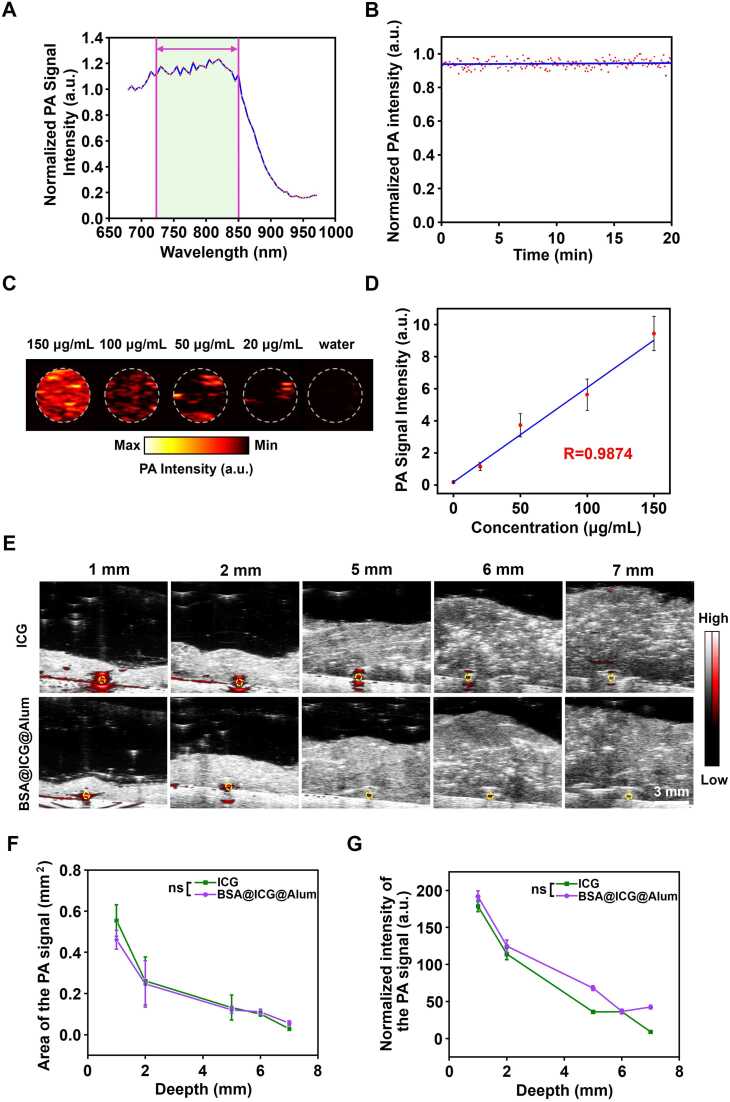
*In vitro* PAI of BSA@ICG@Alum. (A) PA signal intensity of BSA@ICG@Alum at different wavelengths. (B) PA signal intensity of BSA@ICG@Alum in 20 min (λ = 800 nm). (C) PA images of BSA@ICG@Alum with different concentrations (λ = 800 nm). (D) PA signal intensity of BSA@ICG@Alum with different concentrations. (E) PA images of ICG and BSA@ICG@Alum at different depths (λ = 800 nm). (F) PA area of ICG and BSA@ICG@Alum at different depths, ns represents no significant difference. (G) PA signal intensity of ICG and BSA@ICG@Alum at different depths (n = 3), ns represents no significant difference.

The BSA@ICG@Alum exhibited high biosafety, and its PA signal at 680–970 nm was subsequently investigated. The results demonstrated that the PA signal exhibits robust PA properties in the 725–850 nm wavelength range (Fig. 4A), BSA@ICG@Alum has excellent photostability over 20 min (Fig. 4B). *In vitro* PAI indicated the UV-Vis-near-infrared absorption for agar imaging (Fig. S6) with varying concentrations (150 μg/mL, 100 μg/mL, 50 μg/mL, 20 μg/mL, and water), which increased with the increasing concentration of BSA@ICG@Alum (Fig. 4C). A linear correlation between the PA signal and 800 nm (R = 0.9874) was evident (Fig. 4D), indicating the exceptional PA imaging performance of BSA@ICG@Alum.

This study aimed to investigate the *in vivo* tissue imaging capabilities of BSA@ICG@Alum. To this end, the PA signal intensity generated by BSA@ICG@Alum at different depths in situ was compared at 800 nm (Fig. 4E). The integration of PA and ultrasonic imaging modalities enabled the effective detection of signals at tissue depths up to 7 mm, resulting in high-contrast images. Yellow circles represented cross sections of capillary tubes. The area of PA signal intensity in comparison to ICG exhibited a negative correlation with depth, with no significant difference observed (Fig. 4F). Quantitative analysis of the PA images revealed that BSA@ICG@Alum exhibited comparable strength to ICG in situ at the deepest depths (Fig. 4G). In short, PAI offered superior resolution and deeper penetration for BSA@ICG@Alum.

### 3.4 PAI of diverse body transportation *in vivo*

**Fig. 5:**
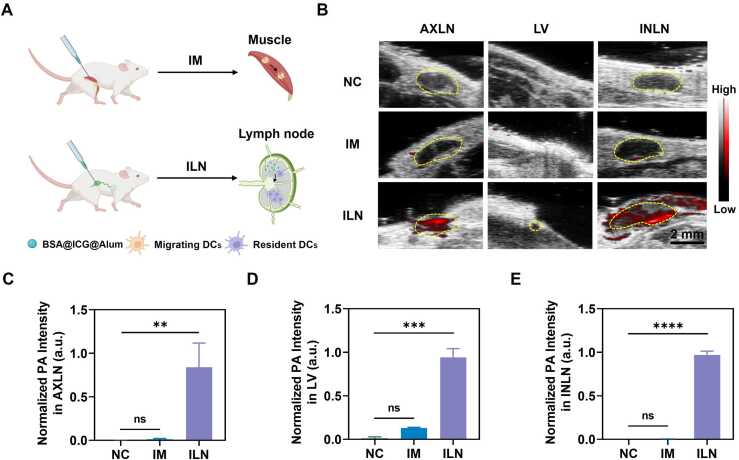
PA images of mice *in vivo*. (A) Schematic representation of mice subjected to intramuscular (IM) and lymph node injection (ILN) with different types of DCs. (B) PA images of AXLN, LV, before, and after intramuscular and lymph node injection in mice subjected to BSA@ICG@Alum, negative control: NC (λ = 800 nm, 30 min later). (C) Normalized PA intensity in AXLN (n = 3), **P < 0.01, ns represents no significant difference. (D) Normalized PA intensity in LV (n = 3), ***P < 0.001, ns represents no significant difference. (E) Normalized PA intensity in INLN (n = 3), ****P < 0.0001, ns represents no significant difference.

BSA@ICG@Alum exhibited excellent immune effects and possessed superior PA properties. To confirm the tracking of the adjuvant, BSA@ICG@Alum was administered by an intramuscular injection and an INLN injection (Fig. 5A), respectively. The interaction of different injection modalities with dendritic cells (DCs) is explored in the later part of the article. The INLN and AXLN were monitored 30 min later. The results (Fig. S7) demonstrated the presence of significant PA signals in the muscle near the intramuscular injection site. However, the periphery of the INLN and AXLN indicated weak signals, and no discernible signals were observed within the LNs (Fig. 5B). This phenomenon may be attributed to the fact that the injected BSA@ICG@Alum initially diffused into the surrounding tissues, and the LNs were not effectively reached. These results further corroborated the antigen storage effect of BSA@ICG@Alum.

In contrast to the intramuscular injection results, BSA@ICG@Alum, which was injected through the INLN, exhibited discernible PA signals in the node and robust PA signals in AXLN, indicating that the injected BSA@ICG@Alum can be transported to the distal LNs through the LVs (Fig. 5B). Furthermore, it demonstrated that BSA@ICG@Alum possessed excellent optical properties. The quantitative results (Fig. 5C, D, and E) demonstrated that, in comparison to the intramuscular injection, the INLN injected with BSA@ICG@Alum exhibited robust PA signals in the AXLN and via LVs. Furthermore, there was a notable increase in the PA signals in the LVs following intramuscular injection in comparison to those observed before injection (Fig. 5D). This demonstrated that following the administration of the adjuvant into the muscle, it traversed the surrounding tissues and entered the lymphatic microvessels, subsequently being transported along the lymphatic system to the regional LNs. However, due to the prolonged nature of this process, the signals detected in the LNs were less pronounced in a relatively short period. On the contrary, lymph node injection results in rapid circulation of lymphatic system manifestations, which offers the potential for developing rapid immunization vaccines. Different modes of injection result in different rates of vaccine adjuvant transport and are critical for adjusting the composition, dosage, or administration of vaccines to improve their efficacy and safety.

To determine whether ICG in BSA@ICG@Alum binds and dissociates from tissue *in vivo*, leading to imaging. Since BSA@ICG@Alum contains aluminium, BSA@ICG@Alum was injected into the INLN. (Fig. S8) 30 min later, the content of aluminium in the AXLN was 0.81 μg higher than that in the control group (no BSA@ICG@Alum injection). The content of aluminium in the INLN was 1.58 μg higher. A significant difference indicated that the BSA@ICG@Alum was efficiently injected into the LNs and can be transported to the distal LNs (AXLN).

### 3.5 3D PAI *in vivo*

**Fig. 6:**
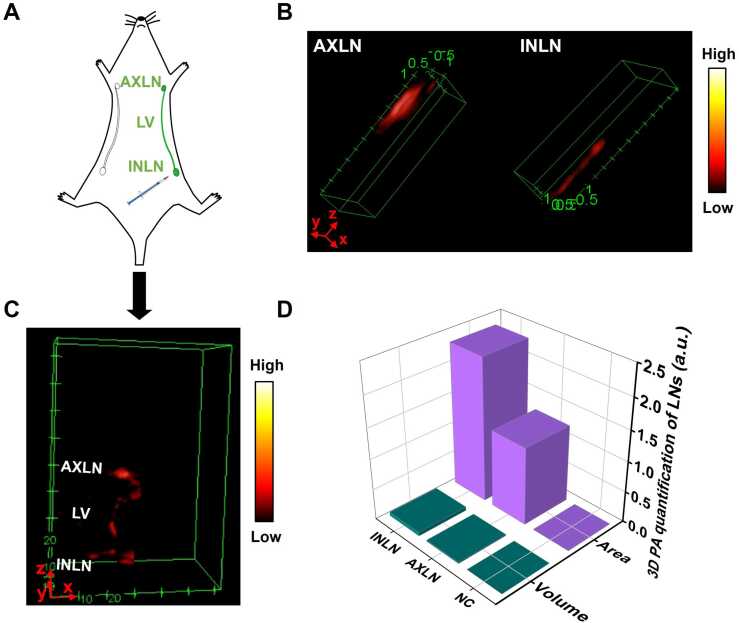
3D PAI body images of mice. (A) Schematic diagram of mice injected with BSA@ICG@Alum. (B) 3D PAI of AXLN and INLN, unit: mm. (C) Alum from INLN to the AXLN of 3D PAI, unit: mm. (D) Volume and surface area quantification of Alum distribution of AXLN and INLN.

To provide a more visual illustration of the transport of BSA@ICG@Alum from the INLN to the AXLN, a schematic diagram of a mouse being injected by the inguinal injection was depicted (Fig. 6 A). Then 3D reconstructions of the INLN and AXLN were presented (Fig. 6B), and then 3D reconstruction of dynamic scanning PA monitoring the BSA@ICG@Alum process from the INLN to the distal lymph node AXLN (Fig. 6 C), which was presented in an intuitive and stereoscopic manner (Video. 1). The ImageJ was used to form a 3D image (Video. 2 and Video. 3) and quantify Alum in AXLN and INLN, and the volume and surface area of Alum in AXLN and INLN were measured (Fig. 6D). The volume of Alum distributed in AXLN and INLN was 0.021 mm^3^ and 0.062 mm^3^, the surface area of Alum distributed in AXLN and INLN was 1.267 mm^2^ and 2.338 mm^2^. Furthermore, the study indicated that PAI offers advantages in terms of in-depth and positioning tracking, while 3D imaging enables quantified visual research on adjuvants.

### 3.6 spatiotemporal PAI *in vivo*

**Fig. 7:**
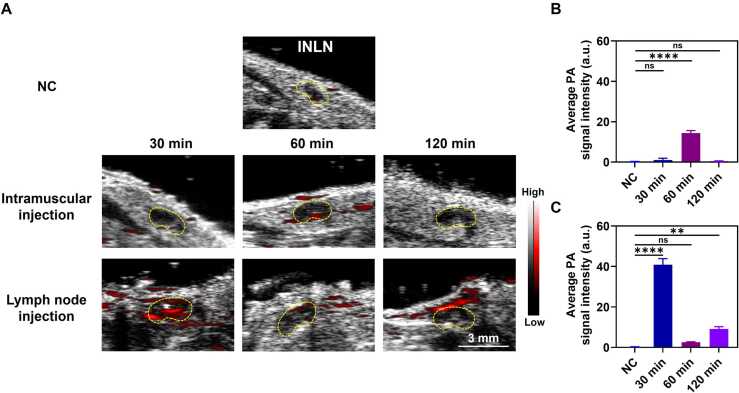
PA spatio-temporal results of BSA@ICG@Alum at INLN. (A) PA images of BSA@ICG@Alum before and 30 min, 60 min, and 120 min after injection. (B) Quantitative PA results after BSA@ICG@Alum intramuscular injection (n = 3), ****P < 0.0001, ns represents no significant difference. (C) Quantitative PA results after BSA@ICG@Alum lymph node injection (n = 3), **P < 0.01, ****P < 0.0001, ns represents no significant difference.

PAI was used to follow and evaluate the transport of BSA@ICG@Alum in the lymphatic system after different injection routes. In PAI observations 30 min after intramuscular injection, the imaging signal within the LNs was not significant, indicating weak short-term transport from the muscle to the draining LNs. In contrast, the INLN was able to rapidly and efficiently transport the Alum to distal LNs, such as the AXLN, after injection. To gain a deeper understanding of the transport of Alum in the lymphatic system after intramuscular injection, in particular its difficulty in rapidly reaching the first site of draining LNs, as well as the storage of adjuvant transport after lymph node injection, the study extended the observation time. The results (Fig. 7A) showed that signals from intramuscular injection were detected to reach the first draining lymph node site at 60 min. However, adjuvants injected directly into the LNs showed a significant PA signal in the INLN (Injection site) at 30 min, which then gradually decreased, but there was a small increase in signal in the epidermal lymphatics at 120 min. This indicated that the micron-sized Alum could flow rapidly with the lymphatic fluid, thereby reducing the likelihood of accumulation in LNs and subsequent obstruction of the lymphatic system. The quantitative results showed that the PA signal for the intramuscularly injected Alum was strongest at 60 min, indicating delivery to the rest of the body through the lymphatic system and the blood circulation after 60 min (Fig. 7B). In contrast, the PA signal for lymph node injection was strongest at 30 min (Fig. 7C), suggesting that the drug begins to be transported along the lymphatic system to the distal LNs as early as 30 min after injection.

**Fig. 8:**
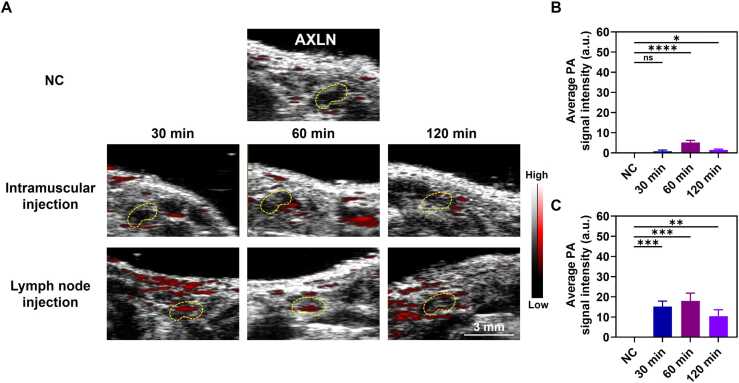
PA spatio-temporal results of BSA@ICG@Alum at AXLN. (A) PA images of BSA@ICG@Alum before and 30 min, 60 min, and 120 min after injection. (B) Quantitative PA results after BSA@ICG@Alum intramuscular injection (n = 3), *P < 0.05, ****P < 0.0001, ns represents no significant difference. (C) Quantitative PA results after BSA@ICG@Alum lymph node injection (n = 3), **P < 0.01, ***P < 0.001.

After intramuscular injection of BSA@ICG@Alum, the distal LNs (AXLN) were similar to the INLN (Fig. 8A, B), and the signals of BSA@ICG@Alum reached the strongest in the AXLN at 60 min after injection, suggesting that this time point is a critical moment for the entry of the Alum from the muscle and the circulation of the Alum in the lymphatic system. There were obvious PA signals in the epidermal LVs and LNs 30 min after lymph node injection, which was consistent with the previous description of 30 min transport to the distal LNs, and the quantitative results (Fig. 8C) showed that the PA signals were strongest at 60 min, indicating that most of the adjuvant could be transported to the distal LNs 60 min after injection, indicating a fast rate of transport, a high volume, and a loss reduction and that 120 min later, the Alum continued to be transported throughout the body by LVs throughout the system. Local concentrations of BSA@ICG@Alum vary over time due to the flow of lymphatic fluid and natural light and sound signal attenuation affecting the PA signal. Therefore, the PA signals of INLN and AXLN weakened at 120 min. Taken together, the results reveal a spatiotemporal transport process of BSA@ICG@Alum in the lymphatic system, especially for the intramuscular route, where 60 min proves to be the critical time point at which the drug begins to diffuse and circulate in the lymphatic system. In addition, the lymph node injection mode promotes more rapid local and systemic distribution of the drug. These PAI results may provide valuable insights for the development of new vaccine delivery systems and lymphatic targeting strategies for therapeutic regimens.

### 3.7 Immunization effects of different modes of administration

The preceding PA results demonstrated that lymph node injection was transported efficiently and effectively to reach the distal LNs. In contrast, the PA signals from intramuscular injection were primarily distributed in the peri-muscular area, which was related to their respective induced immune effects. As illustrated in Fig. 5A, the administration of vaccines into LNs directly activates resident immune cells, particularly DCs, in LNs, thereby enhancing the speed of the immune response. In contrast, intramuscular injection requires the antigenic presentation of migrating DCs to LNs, which induces systemic and long-term immune protection. In response to the diverse individual immune responses to vaccines, some vaccines, such as the influenza vaccine, typically necessitate annual revaccination and provide transient protection. In contrast, the measles vaccine typically confers long-term or even lifelong immunity.

Circle-gate strategies for DCs (Fig. S9A, B) and the results (Fig. S9C, D) demonstrated that the lymph node injection of OVA and OVA/Alum resulted in more DCs in LNs than intramuscular injection. These findings were statistically significant, indicating that lymph node injection facilitates the rapid distribution of the vaccine in the lymphatic system, resulting in a more rapid and robust immune response than intramuscular injection. This observation aligns with the PAI above of transport. Overall, as lymph node injection can cause more DCs recruitment, lymph node injection can reduce the injection dose and induce specific immune responses compared to intramuscular injection. However, lymph node injection can be challenging in terms of technology and promotion of their application, whereas intramuscular injection have a significant acceptance of their application. The actual impact on vaccine formulation and delivery strategies will vary depending on the vaccine's route, composition, and target disease.

## 4 Conclusion

In summary, BSA@ICG@Alum demonstrated multiple beneficial properties, including efficient transport, superior antigen adsorption capabilities, and robust immune response. The assessment of BSA@ICG@Alum verified its safety, stability, and biocompatibility, with no observed damage to tissues or organs post-injection. Moreover, the application of 3D-PAI has elucidated the dynamic transport of BSA@ICG@Alum after lymph node injection in a murine model, particularly within the LVs and LNs, allowing precise quantification of Alum distribution characteristics. The transport of BSA@ICG@Alum was monitored by PAT and it was found that the compound could reach the draining LNs within 60 min of an intramuscular injection, after which it entered the systemic circulation. Alum was observed to be transported effectively to the distal AXLN 30 min following an injection into the INLN, with subsequent transport to the systemic lymphatic system occurring after 120 min. The present investigation elucidates the mechanisms underlying the potentiation of immune responses through the quantification of enhanced recruitment of antigen-presenting cells, predominantly DCs, consequent to lymph node injection. This finding is of pivotal importance for the strategic design of immunotherapies that are tailored to the ever-changing spectrum of antigens. The strategies highlighted in this research effectively address critical challenges, including vaccine efficacy, detection, tracking, and safety. Importantly, this work proposes a new PAI method for tracking, positioning, 3D imaging, and adjuvant quantification, opening new avenues for real-time visualization and monitoring of adjuvant dispersion, which is pivotal in evaluating the success of immunization efforts and shaping strategic vaccine development.

Researchers could, in the future, consider how photoacoustic imaging techniques could assist in the design and optimization of targeted vaccine adjuvants, such as the use of specific molecular markers to label specific cells or tissues, the role of photoacoustic imaging in evaluating adjuvant distribution, dynamics changes, and release patterns, and in understanding adjuvant-induced local and systemic immune responses, and further exploring the potential of photoacoustic imaging in the field of vaccine research and development and the context of research.

## CRediT authorship contribution statement

**Chaohao Liang:** Resources, Investigation. **Barkat Ali:** Supervision. **Jian Zhang:** Writing – review & editing, Supervision, Project administration, Funding acquisition. **Jiarui Chen:** Conceptualization. **Yiqing Zhang:** Investigation. **Changwu Wan:** Supervision. **Fengbing He:** Resources. **Xiaoya Zhao:** Resources. **Bin Yang:** Investigation. **Zhijia Luo:** Methodology. **Lingling Su:** Investigation. **Fan Meng:** Writing – original draft, Visualization, Methodology, Investigation, Data curation.

## Declaration of Competing Interest

The authors declare that they have no known competing financial interests or personal relationships that could have appeared to influence the work reported in this paper.

## Data Availability

Data will be made available on request.
