# Supplementary material for: In vivo spatiotemporal characterizing diverse body transportation of optical labeled high immunity aluminium adjuvants with photoacoustic tomography
Source: Photoacoustics. 2024 Sep 7;39:100643. doi: 10.1016/j.pacs.2024.100643 (PMC11416220; doi:10.1016/j.pacs.2024.100643)
Supplement: Supplementary file 1 — Supplementary material [file mmc1.docx]

**In vivo spatiotemporal characterizing diverse body transportation of optical labeled high immunity aluminium adjuvants with photoacoustic tomography**

Fan Meng^a, b^, Chaohao Liang^b^, Barkat Ali^b^, Changwu Wan^c^, Fengbing He^b^, Jiarui Chen^b^, Yiqing Zhang^b^, Zhijia Luo^b^, Lingling Su^b^, Xiaoya Zhao^d^, Bin Yang^b^, and Jian Zhang^a, b,^ *

*^a^The Key Laboratory of Advanced Interdisciplinary Studies, First Affiliated Hospital of Guangzhou Medical University, Guangzhou Medical University, Guangzhou 510120, Guangdong, China*

*^b^School of Biomedical Engineering, Guangzhou Medical University, Guangzhou, Guangdong 510182, P.R. China*

*^c^School of Chemical Engineering & Technology, China University of Mining and Technology, Xuzhou, Jiangsu 221000, P.R. China*

*^d^School of Pharmacy, Guangzhou Medical University, Guangzhou, Guangdong 510182, P.R. China*

*Corresponding author: jianzhang@gzhmu.edu.cn.


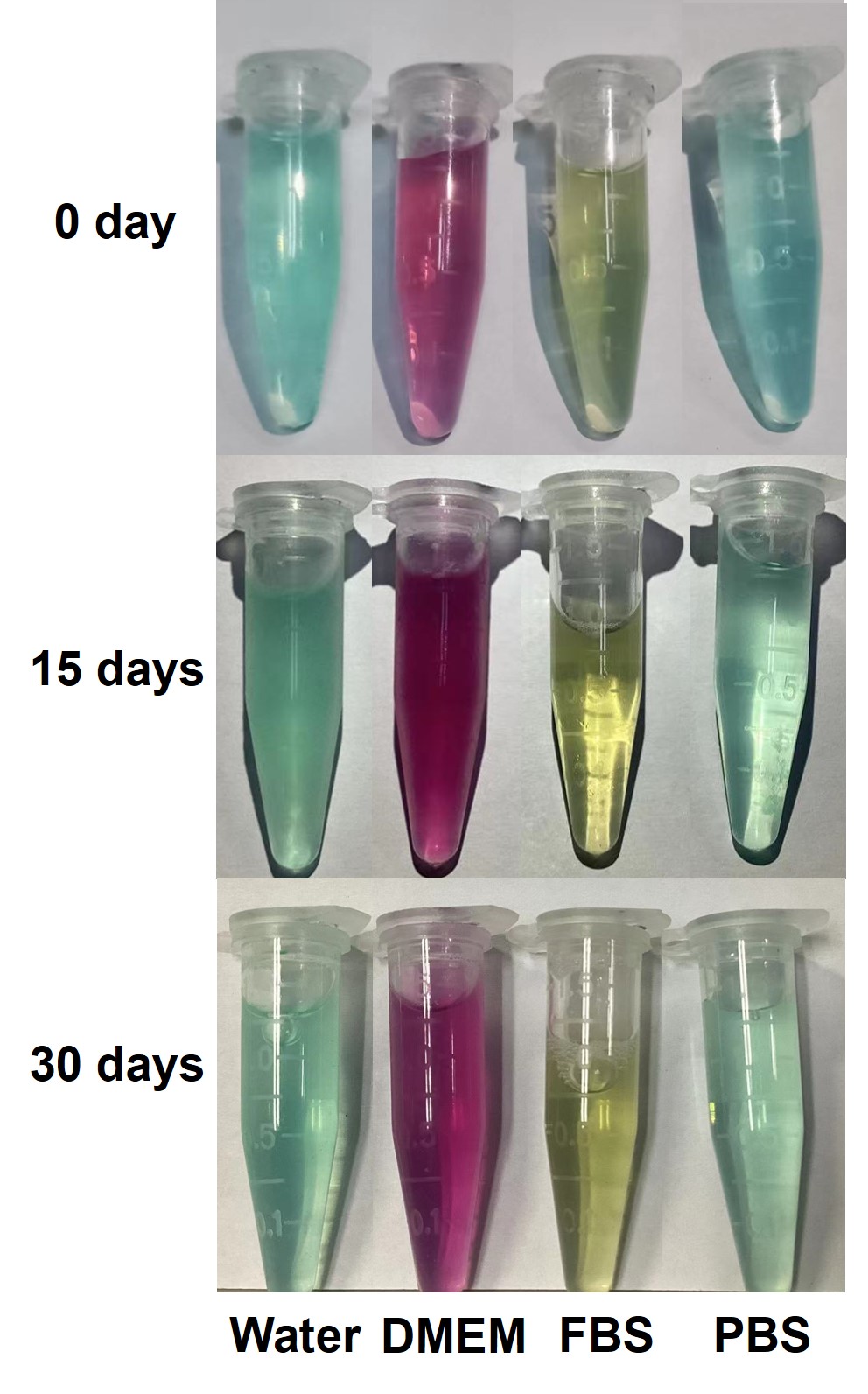


**Fig. S1.** Photographs of BSA@ICG@Alum solutions in diverse solvents following one month of refrigerated storage at 4°C.


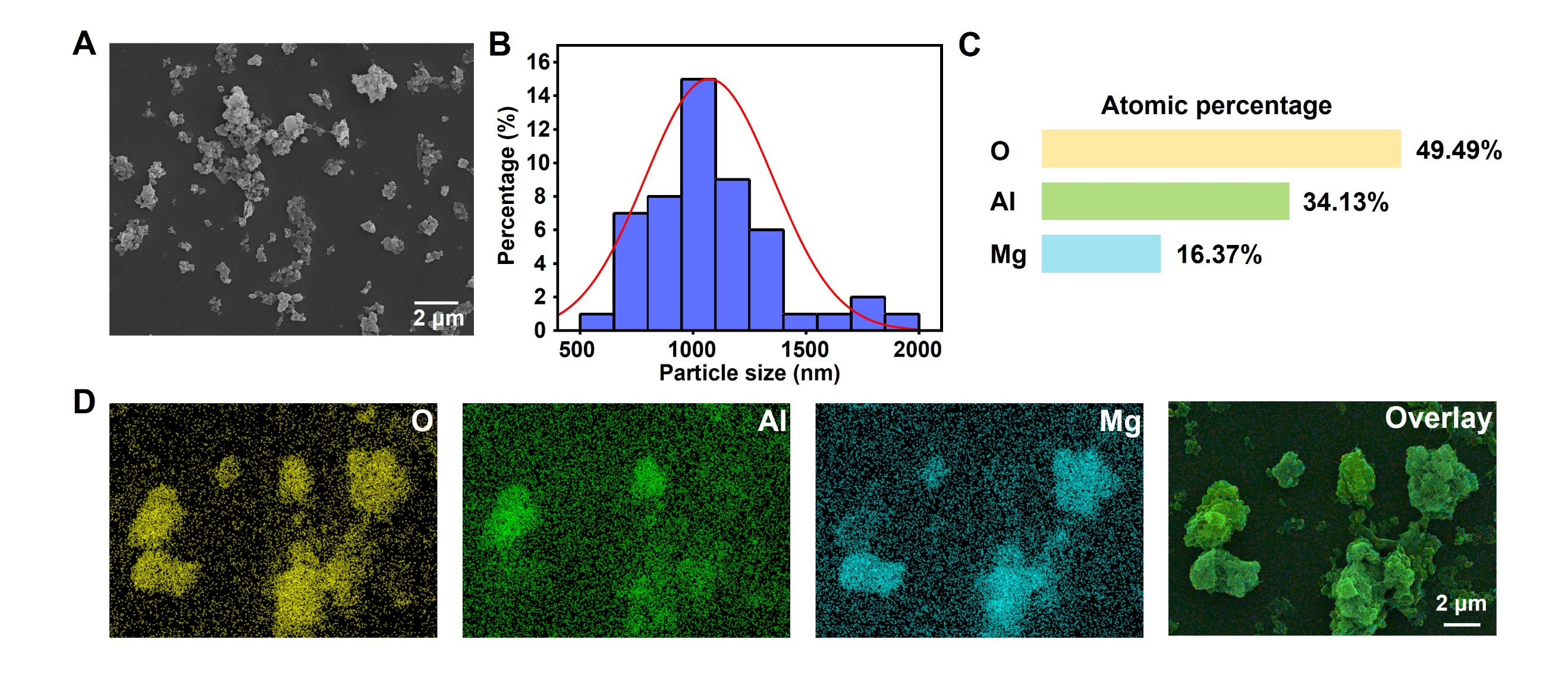


**Fig. S2. Characterization of aluminium adjuvant.** (A) The SEM image of the Alum. (B) The particle size distribution in Fig. A. (C) Different proportions of elements in Alum. (D) EDS indicated the proportions of Alum different elements.


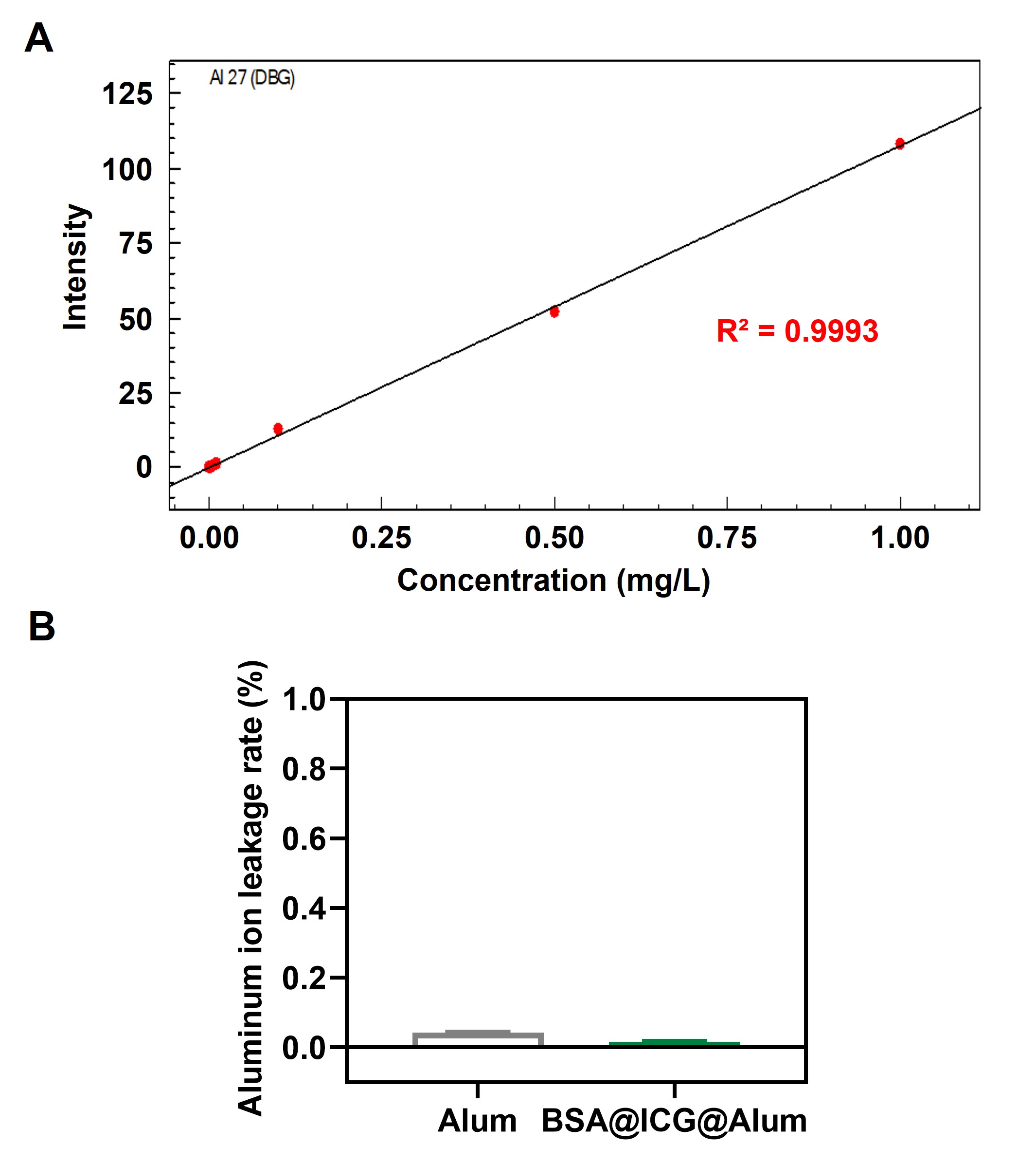


**Fig. S3.** (A) Standard curves for aluminium. (B) Aluminium ion leakage rate of Alum and BSA@ICG@Alum (n = 3).


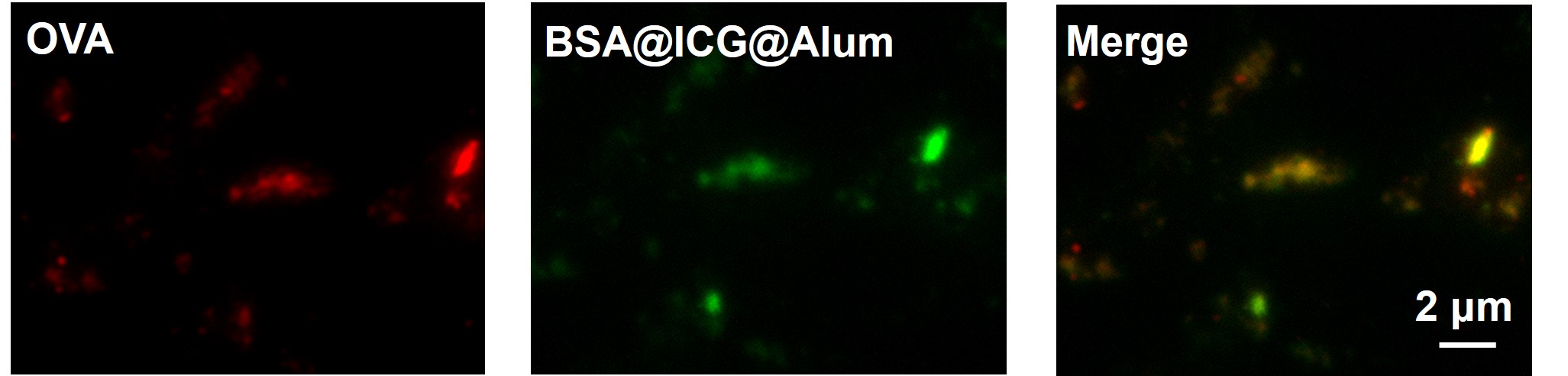


**Fig. S4.** OVA antigen adsorption with BSA@ICG@Alum under 100◊ oil mirror, Cy5@OVA (red), lumogallion-labeled BSA@ICG@Alum (Green), OVA and BSA@ICG@Alum coincide (Yellow).


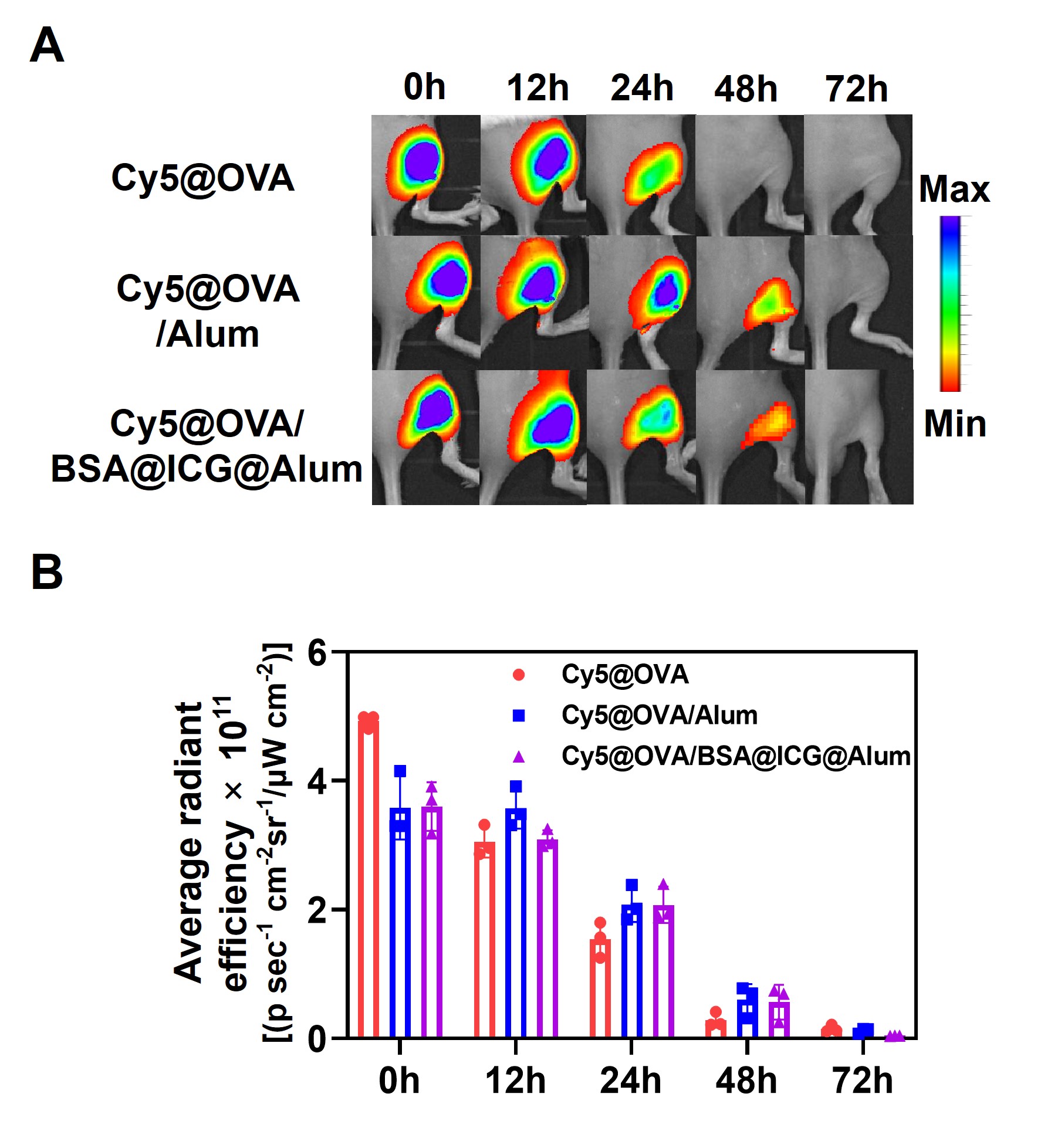


**Fig. S5. Antigen reservoir effect.** (A) *In vivo* images and (B) The mean fluorescent intensity of Cy5-OVA persistence at the injection sites (n = 3).


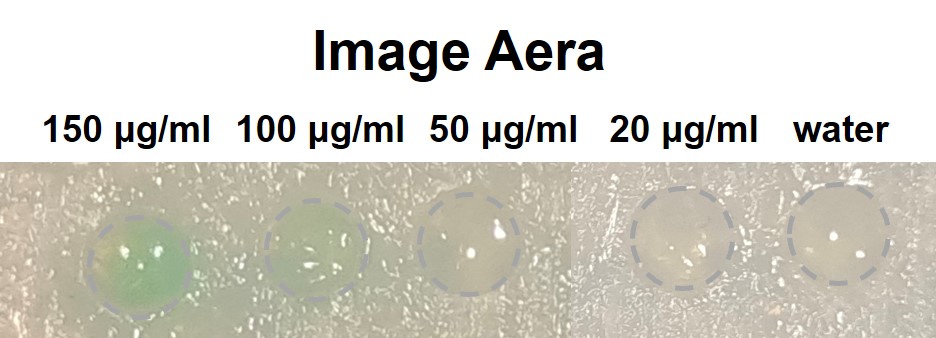


**Fig. S6.** Sample photo of photoacoustic image of BSA@ICG@Alum.


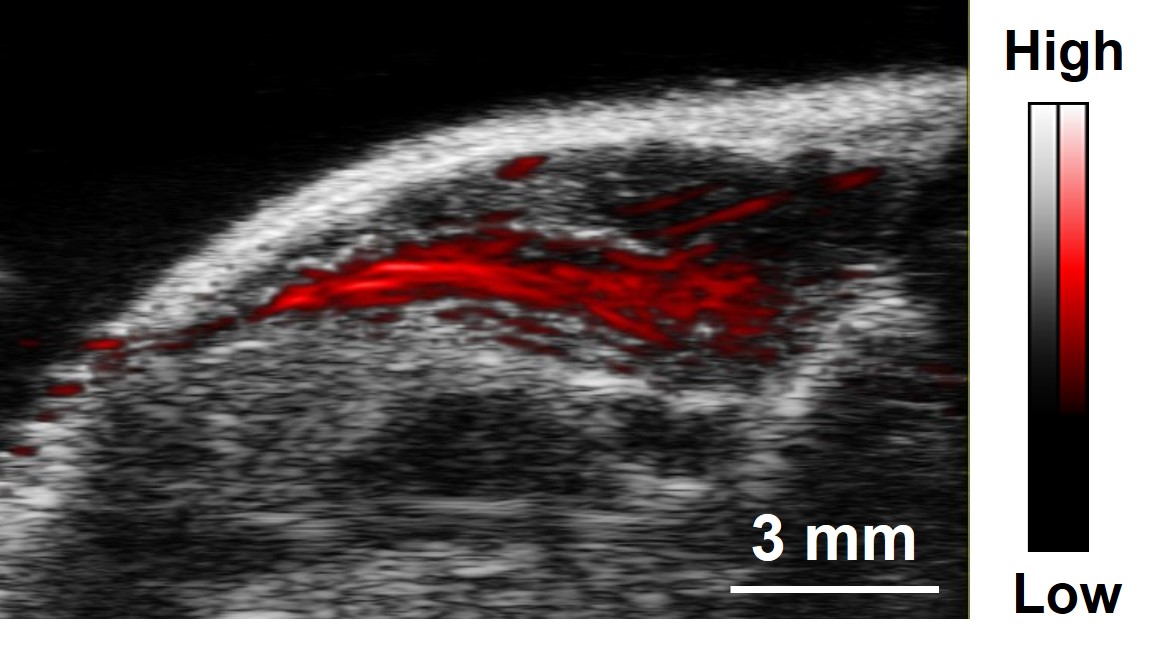


**Fig. S7.** Photoacoustic image of intramuscular injection in situ.

**
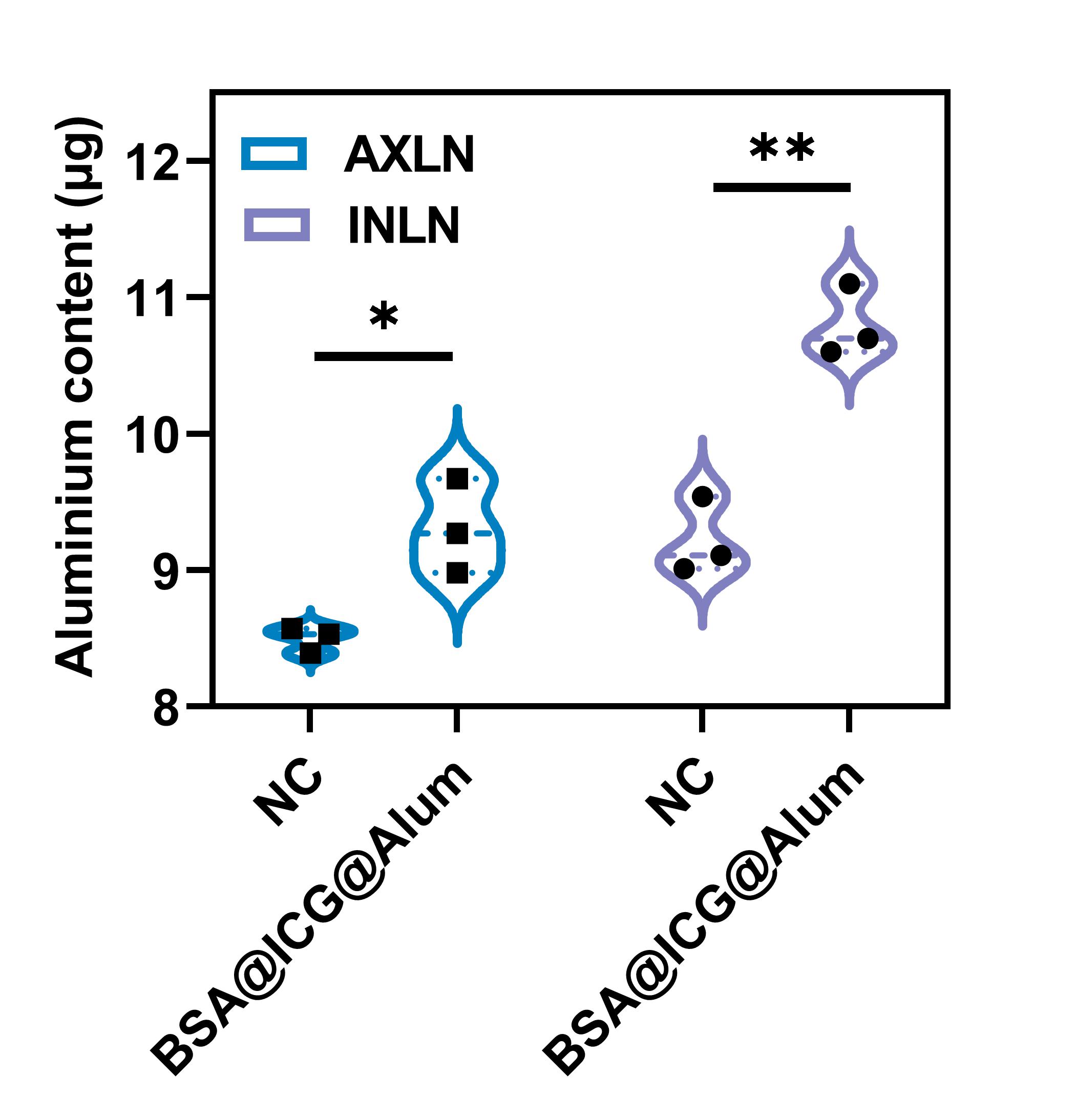
**

**Fig. S8.** Aluminium content in AXLN and INLN (n = 3).


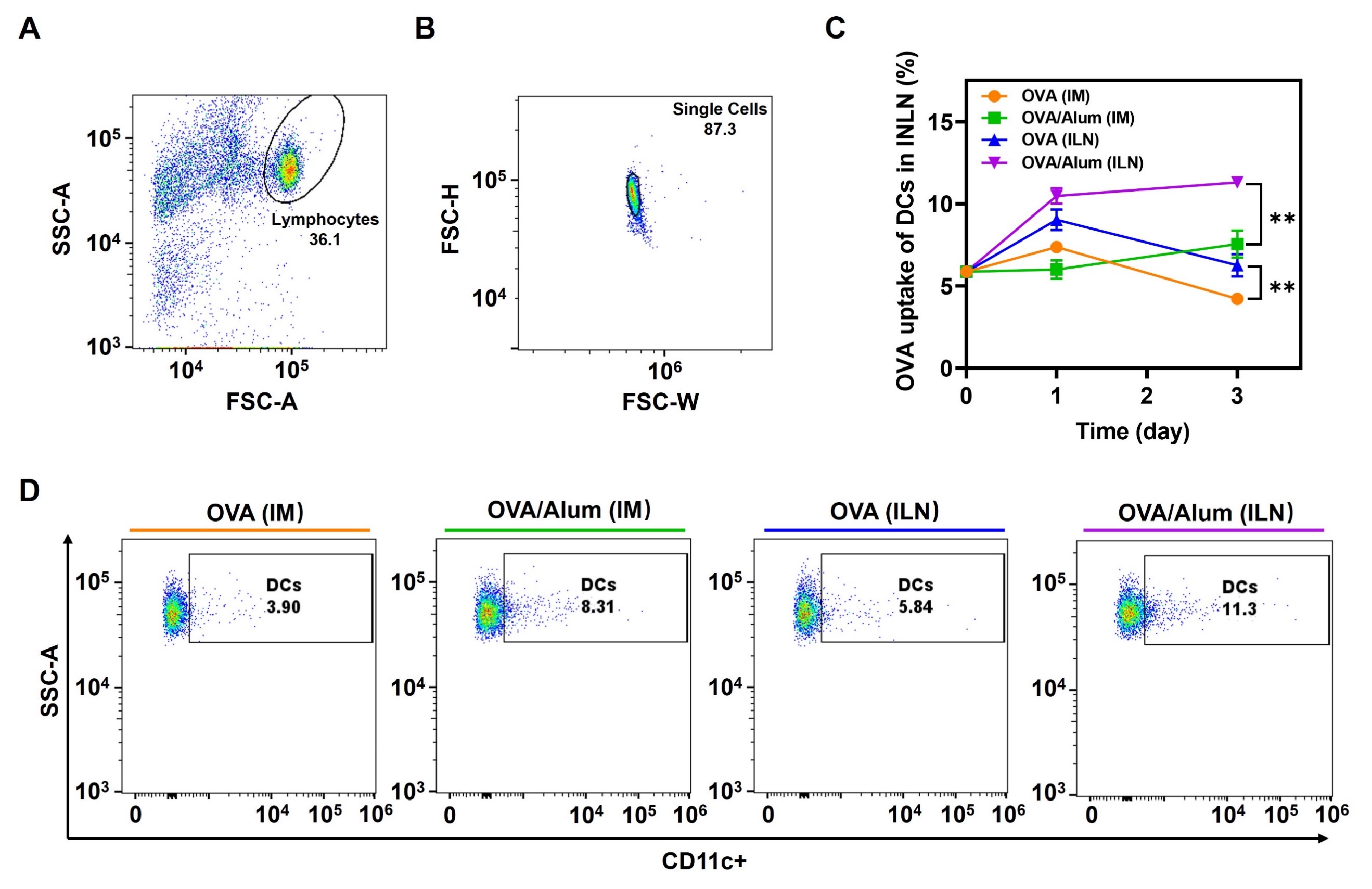
 **Fig. S9. Immune capacity of different injection methods.** (A) Lymphocyte circle-gate strategy. (B) Circle-gate strategy for single cells. (C) The proportion of DCs in LNs of mice after injection. Data were shown as mean s.e.m (n = 3), **P < 0.01. (D) Four types of flow cytometry, including OVA (IM), OVA/Alum (IM), OVA (ILN), and OVA/Alum (ILN).

**Online Video Captions**

**Video. 1. 3D-PAI of inguinal and axillary lymph node in mice, unit: mm.**

**Video. 2. 3D imaging of the axillary lymph node, unit: mm.**

**Video. 3. 3D imaging of the inguinal lymph node, unit: mm.**
